# A Polycrystalline Pd Surface Studied by Two-Dimensional Surface Optical Reflectance during CO Oxidation: Bridging the Materials Gap

**DOI:** 10.1021/acsami.3c11341

**Published:** 2023-12-18

**Authors:** Sebastian Pfaff, Alfred Larsson, Dmytro Orlov, Lisa Rämisch, Sabrina M. Gericke, Edvin Lundgren, Johan Zetterberg

**Affiliations:** † Combustion Research Facility, Sandia National Laboratories, 7011 East Ave, Livermore, California 94550, United States; ‡ Division of Synchrotron Radiation Research, Lund University, Sölvegatan 14, S-22363 Lund, Sweden; § Division of Mechanics, Materials and Component Design, Lund University, Ole Römers väg 1, S-22363 Lund, Sweden; ∥ Combustion Physics, Lund University, Sölvegatan 14, S-22363 Lund, Sweden

**Keywords:** microscopy, *operando* catalysis, mass spectrometry, palladium, polycrystal, surface orientation library

## Abstract

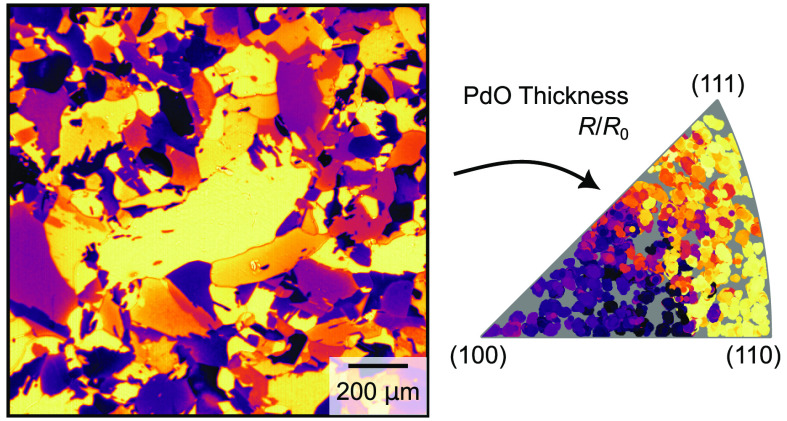

Industrial catalysts are complex materials systems operating in harsh environments. The active parts of the catalysts are nanoparticles that expose different facets with different surface orientations at which the catalytic reactions occur. However, these facets are close to impossible to study in detail under industrially relevant operating conditions. Instead, simpler model systems, such as single crystals with a well-defined surface orientation, have been successfully used to study gas–surface interactions such as adsorption and desorption, surface oxidation, and oxidation/reduction reactions. To more closely mimic the many facets exhibited by nanoparticles and thereby close the so-called materials gap, there has also been a recent move toward using polycrystalline surfaces and curved crystals. However, these studies are limited either by the pressure or spatial resolution at realistic pressures or by the number of surfaces studied simultaneously. In this work, we demonstrate the use of reflectance microscopy to study a vast number of catalytically active surfaces simultaneously under realistic and identical reaction conditions. As a proof of concept, we have conducted an *operando* experiment to study CO oxidation over a Pd polycrystal, where the polycrystalline surface acts as a collection of many single-crystal surfaces. Finally, we visualized the resulting data by plotting the reflectivity as a function of surface orientation. We think the techniques and visualization methods introduced in this work will be key toward bridging the materials gap in catalysis.

## 1 Introduction

Thermal and electrochemical catalysis are key to sustainability.^1^ However, industrial catalysts are complex materials systems operating under harsh conditions. The active parts of an industrial catalyst are nanoparticles that expose a variety of facets with different surface orientations at which the catalytic reactions occur. These nanoparticle facets are nearly impossible to study under the conditions in which the catalysts are commonly used. Instead, *operando* measurements have mainly been performed on more simple low-index single-crystal surfaces. Typically, the idea is to use the results of such measurements to model individual nanoparticle facets, which enables us to piece together the full behavior of a nanoparticle, given that the surface orientations it exhibits are known. This approach has been a cornerstone of fundamental catalysis research in the past decades.^2,3^

With CO oxidation into CO_2_ being used as a model reaction, many *operando* studies of CO oxidation have been conducted on the low-index (100), (110), and (111) surfaces of transition metals. These have been described extensively in terms of both their oxide formation properties and their catalytic activities. In addition to this, studies have been performed on high-index stepped or kinked surfaces. Such studies have proven a clear correlation between the surface orientation and the catalytic properties of said surface.^4−10^

However, most of these studies have focused on only one or a rather limited subset out of all possible surface orientations. This approach requires many consecutive experiments and many catalytic samples to fully probe the surface orientation space. Furthermore, the use of multiple samples makes it very difficult to ensure consistent gas conditions throughout the experiments, as each sample will change the gas conditions depending on its activity;^11^ a more active sample will inevitably have more product gas and less reactant gas in its gas boundary layer.

In the past decade, polished polycrystals have been proposed as a solution to this problem. These crystals, which consist of a large number of randomly oriented grains, will, when polished, exhibit a wide variety of surface orientations. Therefore, an increasing number of studies have been performed in which two-dimensional (2D) techniques are used to study polycrystalline samples.^12−15^ With appropriate 2D resolution, these polycrystals now act as a collection of a vast number of separate single crystals, all within one sample.^16^ In this way, multiple surface orientations can be measured simultaneously, which is much more efficient and also makes it easier to compare results, as we can expect similar experimental conditions between neighboring grains. The size of the grains and thus the number of orientations per surface area can also be tuned by changing the manufacturing process of the polycrystals.

Studies with polycrystals are often conducted by first mapping the orientations of grains within a region of interest (ROI) on the sample using electron backscatter diffraction (EBSD). The same ROI is then investigated using 2D-capable techniques such as photoemission electron microscopy (PEEM)^17^ or scanning photoelectron microscopy (SPEM).^13^ A number of individual grains in the ROI are then chosen, and the properties of these grains are examined in detail. Unfortunately, in these studies, the grains are treated more as individual data points rather than as a quasi-continuous set of single-crystal surfaces covering a vast number of surface orientations. Another caveat is that the PEEM and SPEM techniques can also operate only under low-pressure conditions.

2D-surface optical reflectance (2D-SOR) as a technique to study catalysts was introduced by Onderwaater et al.,^18^ showing that a simple setup measuring the optical reflectance of a metal sample can be used to obtain information about the sample oxidation or roughness. The technique has also been used to study corrosion-related phenomena.^19−21^ Further experiments have shown that even very thin oxides with a thickness of only a few nanometers can be detected.^22^ We have since further developed this method and have also used it in combination with high-energy surface X-ray diffraction (HESXRD), where we correlate changes in the surface reflectance with changes in the surface oxide thickness and roughness on a single crystal Pd(100) sample.^14,23,24^ It turns out that the 2D-SOR signal is sensitive enough to detect the formation of a 2–3 Å thick surface oxide^25^ on Pd(100). Especially when combined with other *operando* techniques, such as mass spectroscopy (MS) or PMIRRAS,^26^ 2D-SOR can help correlate changes in surface structure with changes in chemical activity. Another advantage of 2D-SOR is its high time resolution, which is primarily limited by the camera used to image the reflectance rather than by the sample itself. This allows for repetition rates in the order of the speed of the gas diffusion over the sample under atmospheric conditions.^11^ Furthermore, 2D-SOR can operate at any pressure, as opposed to the electron-based 2D experimental techniques mentioned above.

In this work, we combine EBSD and 2D-SOR to characterize a polycrystalline sample surface in an *operando* study. Previously we reported on the potential of this approach.^14^ In this article, we exploit previous progress to explore a much larger data set and report on variations in the thickness of PdO formation on different surface orientations. However, instead of selecting a number of grains in the ROI and treating them individually, we treated the grains as a massive collection of data points. This approach provides new information under operation conditions of a multifaceted catalyst at work, which has not been performed previously and creates further challenges in how to efficiently present the data. In ref (13) the concepts of the step edge parameter (SEP) and the step density parameter (SDP) are devised, which describe the surface in two simple variables. We chose to plot our data against both of these variables. Furthermore, we visualize the data in a way that does away with the traditional spatial representation of the polycrystal by plotting the reflectivity as a function of the surface orientation using the so-called inverse pole figure (IPF), which is another representation of the surface orientations commonly used in crystallography.^27^ This way of visualizing data is very useful to draw conclusions regarding how the surface orientation affects surface reactivity and has been used previously to visualize data as a function of the surface orientation.^28,29^ It also shows the strength of the 2D-SOR technique in quickly obtaining large amounts of 2D-surface information in *operando* catalysis experiments.

As a proof of concept, we conducted an *operando* experiment under near-ambient pressure conditions in which we track the reflectivity of the grains of a Pd-polycrystal performing CO oxidation in an oxygen-rich environment. We then link the reflectivity changes to the surface oxidation. Even though we only present data from this one experiment, the setup and principle presented herein can easily be adapted to other samples and reaction environments, such as the solid–liquid interface in electrochemical experiments.^30,31^

## 2 Experiment

In this experiment, we used a hat-shaped Pd polycrystal with a bottom diameter of 8 mm, a top diameter of 6 mm, and a height of 2 mm purchased from SPL in Zaandam. The sample was polished (ra <0.03 μm) and had a specified purity of 99.994%. Before the measurements, the surface was cleaned by three cycles of Ar^+^ sputtering and annealing at 1000 K. The sample was transferred through air between the sputtering and measurements. When choosing what polycrystal to use, the size of the grains is important. The size has to be large enough to obtain good per-grain statistics while small enough to minimize gas gradient effects within the region of interest (ROI) as it is desirable that all grains experience the same gas conditions. For more discussion on this, see ref (11). The sample used has grains of the order of 10–100 μm in size, which is small enough to assume that all grains experience the same overall gas conditions.

**Figure 1:**
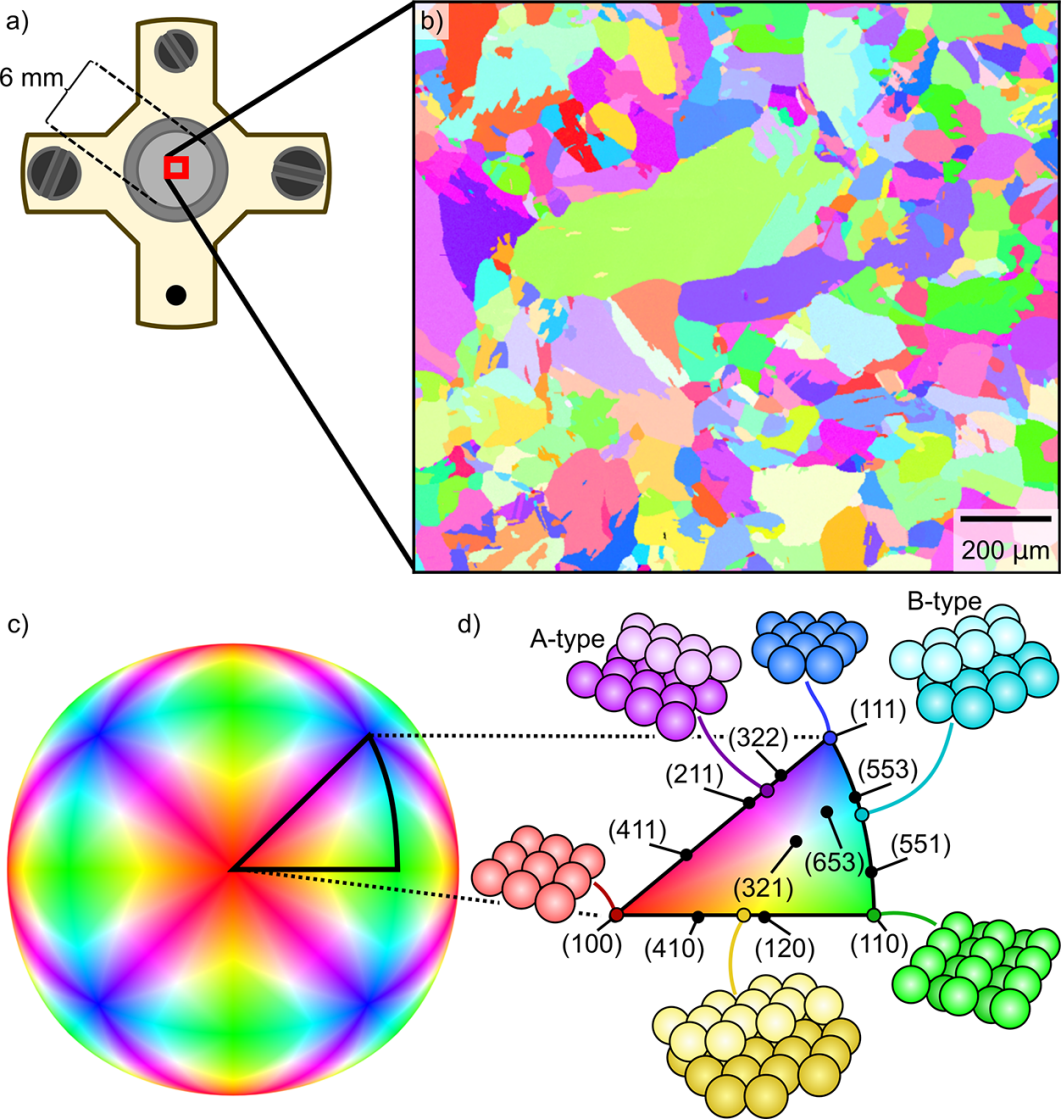
Surface orientations of the grains on the sample. Panel a shows the region of interest (ROI) on the 6 mm diameter top hat-shaped sample, while panel b shows the grain orientation map, also called IPF map, of the ROI as measured by EBSD. Panel c shows the origin of the IPF color map used to represent the orientations as a part of a sphere which is colored to match the rotational symmetry of the unit cell. Panel d shows how the colors and locations in the IPF map to various Miller indices and the resulting surface terminations, assuming a perfect cut with no reconstructions or refaceting.

The crystallographic orientations of the grains were characterized by electron backscatter diffraction (EBSD) using a scanning electron microscope (FEI Quanta 200 MKII) with an integrated camera (Hikari XP) and a TSL-OIM system from EDAX. In this way, we surveyed a ROI of 1.43 × 1.26 mm^2^, which will be the area of the sample used in this work as shown in Figure 1a. The ROI was chosen to be approximately in the center of the sample, but the exact choice of the area to use was arbitrary. Figure 1b shows the orientation map of the grains in the ROI of the sample. Here it should be made clear that EBSD is a bulk technique—the determined orientations and Miller indices of the surfaces assume a perfect cut of the bulk grains with no refaceting or restructuring.

**Figure 2:**
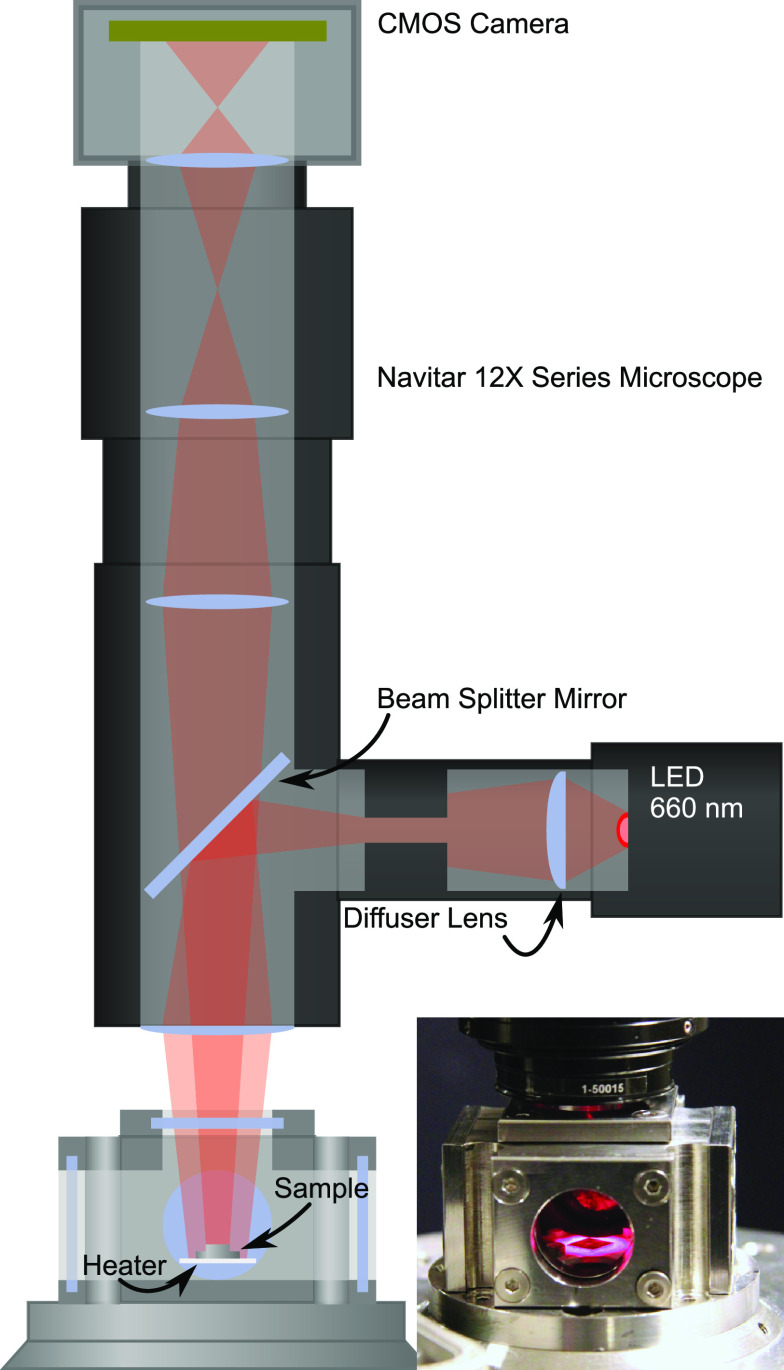
Schematic of the experimental setup. The reflectivity of the sample surface is measured by shining 660 nm LED light on the sample through a beam splitter. The reflected light is then imaged through a microscope and recorded using a CMOS camera. The inset photograph shows the reactor with a 4 × 4 mm^2^ sample as a reference. Reproduced with permission from ref (14).

The 2D-SOR microscope setup shown in Figure 2 consisted of off-the-shelf parts. The main part of the microscope is a preassembled lens system (Navitar 12X Zoom Series) with an optical illumination port. At this port, a high-intensity red LED at 660 nm (Thorlabs M660L4) is attached, which acts as a light source. A diffuser lens placed between the LED and the beam splitter removed any patterns from the LED itself. The light was reflected off the sample and imaged using a 16-bit Andor Zyla camera. This provides a flexible, portable, and inexpensive system that can easily be mounted on any reactor with optical access. The setup is described in more detail in ref (14).

**Figure 3:**
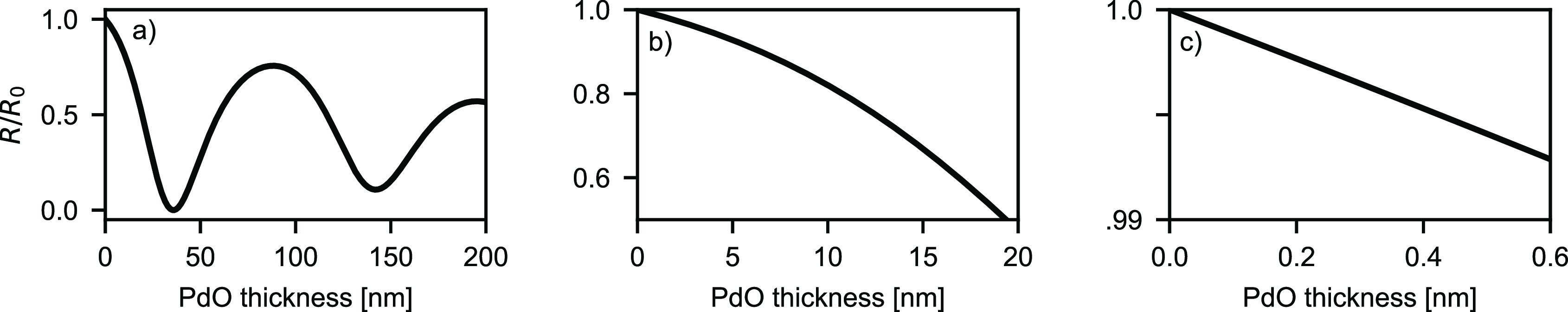
Sample reflectivity as a function of PdO thickness. Panels a–c all show the same data, but are increasingly zoomed in to the area around zero PdO thickness. The model used to calculate these reflectivities is dependent on the refractive indices of PdO, the light wavelength, and the incident angle. It is also assumed that the surface does not exhibit any roughness and that the light incidence is normal to the surface.

To quantify the reflectance data, we refer to the Fresnel equations and roughness calculations as discussed thoroughly in ref (22). In the calculations, we use the optical constants for bulk Pd metal and bulk PdO.^32,33^ Further assuming negligible surface roughness, we can find the oxide thickness from the loss in reflectivity compared with the reduced surface without any oxide. This is illustrated in Figure 3.

The experiment was performed in a 23 mL high-pressure flow reactor. Optical access to the sample was provided by 18 mm diameter windows on all sides. Sample heating is done with a Boralectric resistive heater, onto which the sample is placed. The temperature of the sample was monitored with a type D thermocouple connected to the heater. Calibration measurements map the temperature reported by the thermocouple to the real sample temperature as discussed in ref (34).

The gas supply into the reactor is regulated with a series of mass flow controllers (Bronkhorst EL-FLOW), and a pressure controller (Bronkhorst EL-PRESS) is used to keep a constant pressure in the reactor. Using this system, we can reach flows between 10 and 500 mln/min at pressures between 10 mbar and 1 bar. Pressure gauges monitor the pressure before and after the reactor, which makes it possible to determine the reactor pressure through a calibration curve. A quadropole mass spectrometer (Pfeiffer QMP 220) into which a small amount of the exhaust gas was leaked through a leak valve was used to monitor the gas composition at the reactor outlet. More details on the reactor, the gas system, and its capabilities can be found in refs (24, 34, and 35).

### 2.1 Data Visualization

In addition to showing the reflectivity images themselves, we have chosen to present the results in two ways: First, we plot the value of the reflectivity of a grain in the IPF. Because we are working with Pd, which has a cubic lattice structure, there is a 6-fold rotational symmetry in the unit cell. Thus, each surface orientation is assigned both a unique color and a unique position in the IPF, as shown in Figure 1b–d.^36^ Now we can use other data, in this case reflectivity data, and replace the color of the corresponding grain in the IPF with the reflectivity data while keeping the position. In this way, we can summarize the entire data set into an easy-to-understand form where we can plot a parameter, in this case the reflectivity, as a function of the grain orientation. The second method to present the data is by plotting the reflectivity of each grain as a function of the SEP and SDP, which have been devised in the work by Winkler et al. to quantify surface orientation properties into two attributes.^13^

## 3 Results and Discussion

### 3.1 Experimental Results

To demonstrate the approach of combining a 2D-capable technique with an orientation mapped polycrystal, we performed an experiment on CO oxidation under oxygen-rich gas conditions. The sample was heated in a mixture of 40% O_2_, 4% CO, and 56% Ar at a pressure of 150 mbar and a total flow of 100 mL/min. The sample surface reflectivity was monitored using a 2D-SOR microscope at an image acquisition rate of 50 Hz. The sample temperature was gradually increased from room temperature to around 450 °C.

**Figure 4:**
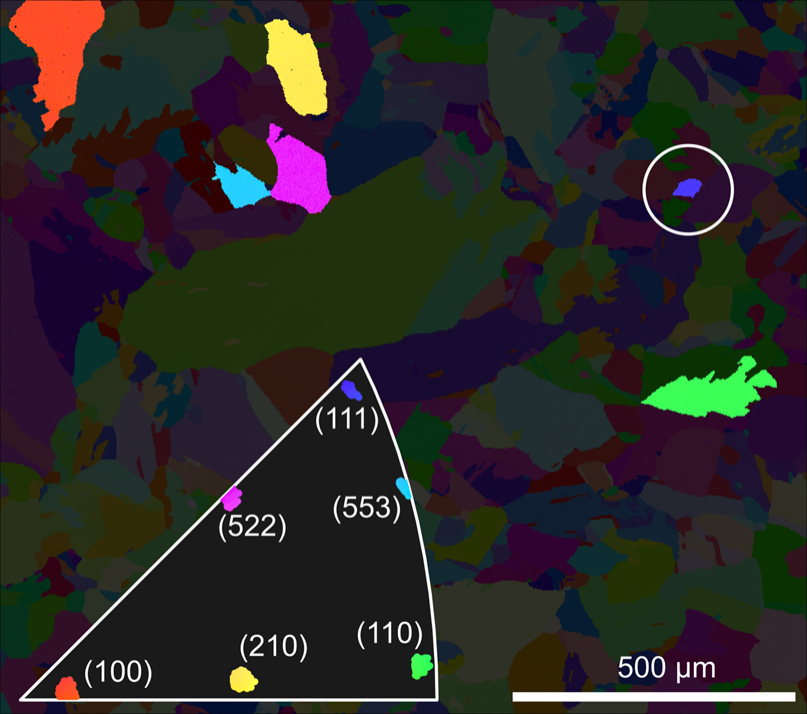
Grains whose reflectivity trend is shown in Figures 5 and 6 along with their approximate Miller indices. These grains correspond to representative surfaces which are also discussed in more detail in the Discussion section.

**Figure 5:**
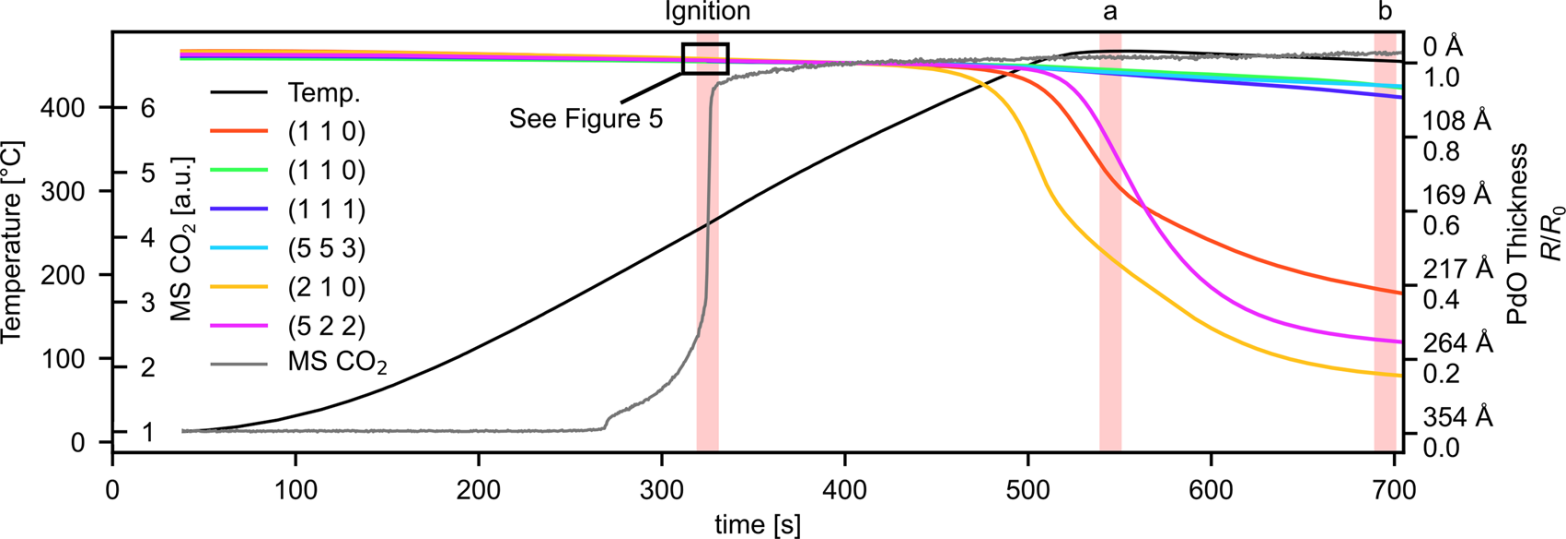
Overview of the CO oxidation experiment. The black trend shows the sample temperature over time while the gray trend shows the CO_2_ concentration in the reactor, which corresponds to sample activity. We observe that the sample ignites at around 320 s. The small increase at 280 s is attributed to carbon desorbing from the heater. The colored trends show the change in reflectivity for the grains highlighted in Figure 4. The areas highlighted in red correspond to the time when the data shown in Figures 6 and 7 were recorded.

This section will give a short overview of the results, which will then be discussed in more depth in the Discussion section. To begin with, we examine the reflectivity of six representative grains as highlighted in Figure 4. Figure 5 shows the partial pressure of CO_2_ and the sample temperature during the experiment as well as the reflectivity trends of the highlighted grains. As the sample is heated, the CO_2_ production increases exponentially until the reaction reaches the mass-transfer limit (MTL) at around 200 °C. This event is also known as the catalytic ignition.^37^ The small increase in the level of CO_2_ at 280 s is attributed to carbon desorbing from the heater. It should be noted at this point that this means that all grains ignite more or less simultaneously—more on that later. This article focuses on the three time ranges indicated in Figure 5, the first of which is the aforementioned ignition. The other two, denoted a and b, are with the sample in a highly active state. The reflectivity map in each of these ranges has been normalized with an image of the clean metallic sample.

**Figure 6:**
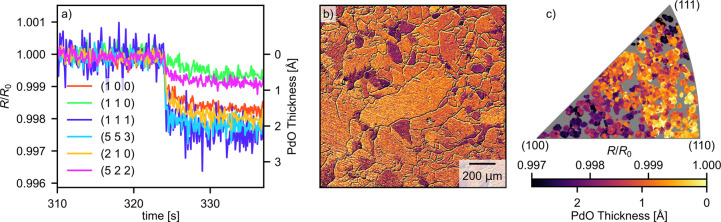
Sample behavior at the catalytic ignition. Panel a shows the reflectivity trends for the grains highlighted in Figure 4. Panel b shows the relative reflectivity change at the ignition for the chosen ROI on the sample, while panel c shows the same data plotted in the IPF as a function of the surface orientation.

The change in the reflectivity of the sample surface at the ignition is shown in Figure 6. Panel a shows the development of the reflectivity of the grains highlighted in Figure 4 during the catalytic ignition. Here we observe a very small decrease in the reflectance of around 0.3% for some grains. Panel b shows the reflectivity of the sample in the ROI, and panel c shows the same data, but plotted in the IPF. We observe that in particular the grains close to the (111) and (100) orientations exhibit a clear drop in reflectance.

**Figure 7:**
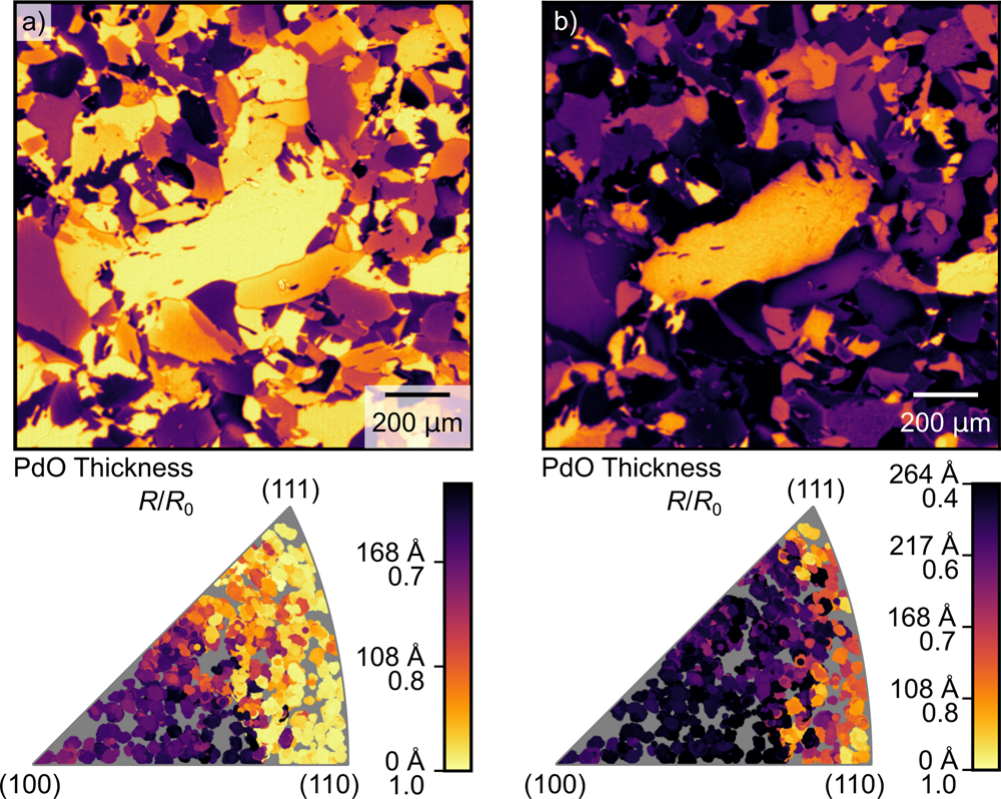
Reflectivity of the sample when a thick oxide has formed. Panels a and b show the reflectivity at points a and b in Figure 5, respectively. Note that the color scale here is very different from that in Figure 6. We observe that in (a), primarily the grains toward the (100) orientation have oxidized, with the thickest oxide having formed on grains with the (210) orientation. Later, in (b), more grains have formed a thick oxide.

The changes in reflectivity later in time, in regions a and b, are shown in Figure 7. Here, the surface is in the MTL regime, while the temperature has been increasing. Panels a and b show the reflectivity in regions a and b, respectively. We observe a significant decrease in the surface reflectivity across most grains except those close to the (111) and (110) orientations. This increases as the sample is further heated. Note the difference in color scale between panels a and b. In the Supporting Information, a video showing the oxidation of the sample during the entire experiment is provided.

**Figure 8:**
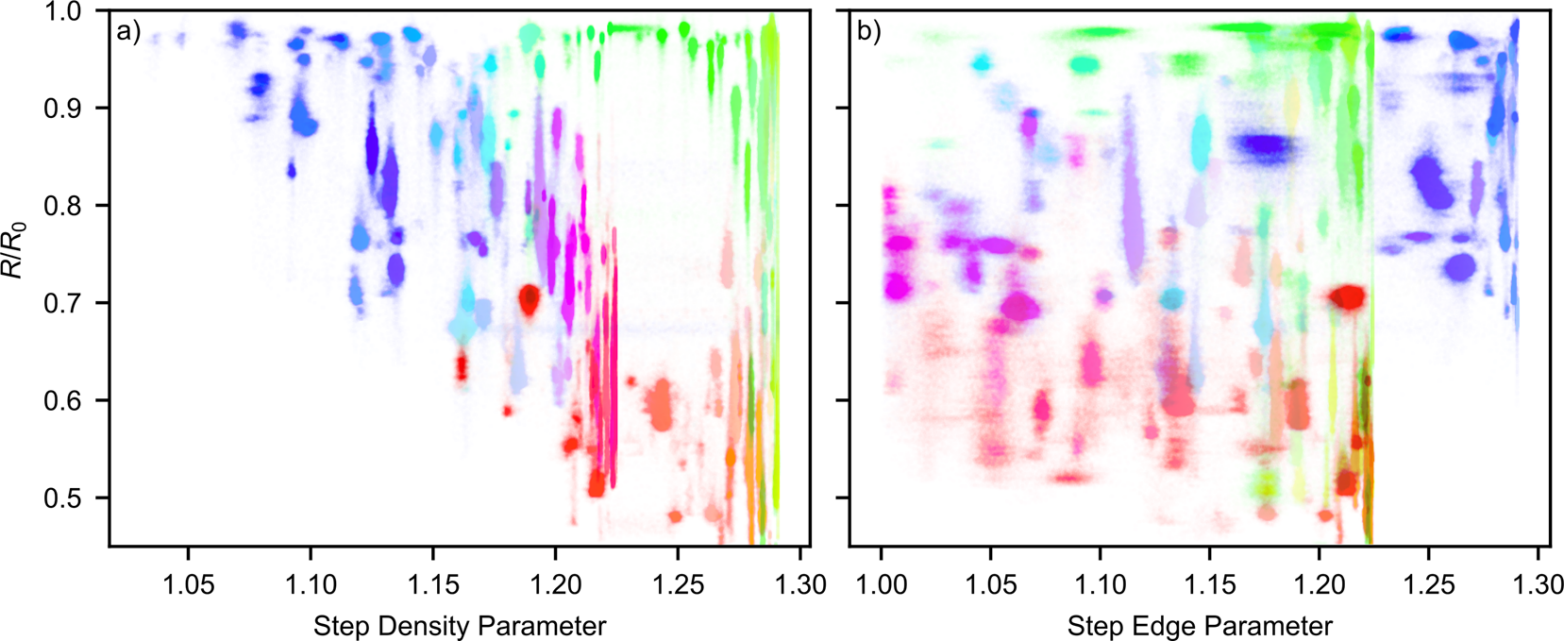
Reflectance data from region a in Figure 5 plotted against (a) the SDP and (b) the SEP, as defined in ref (13). A higher reflectance value corresponds to a less oxidized surface. The colors of the points correspond to the colors assigned to that surface orientation in the IPF map of the sample surface.

By calculating the SDP and SEP for every pixel in the image, based on the EBSD data, we can also plot the reflectance data in region a as a function of the SDP and SEP. This is shown in Figure 8.

### 3.2 Discussion

Comparing the results in this study to existing literature is not an easy task due to the very large number of surfaces available. In this discussion we will focus on the behavior of six representative grains with orientations close to the (100), (110), (111), (553), (522), and (210) orientations as shown in Figure 4. First, we can conclude that the surface orientations on the left edge of the IPF have A-type steps, whereas those on the right edge have B-type steps. All areas in between will have a mixture of both types of steps, with different ratios depending on the position in the IPF.

Most studies on catalytic CO oxidation on Pd agree that as the sample is heated, it “ignites” whereby it transitions from an inactive stage of CO poisoning where the surface is covered by CO to a stage where the sample is active and mainly covered in adsorbed oxygen or Pd oxides.^38^ The ignition temperature depends not only on the surface structure but also on the gas conditions and is further affected by the coupling between the gas and the surface.^11,39^ At this point, the activity also reaches the MTL, where it is limited by the diffusion of the reactant gases to the surface. As the sample is heated further, bulk oxides may form, which in the case of Pd may also be catalytically active.^40^ The following discussion will be split into two parts, discussing the thin oxide formation at the ignition and the subsequent thicker oxide separately.

#### 3.2.1 Ignition

We begin by comparing the results of this study with the literature concerning low-index Pd surfaces, which have been studied extensively. These can be found in the corners of the IPF.

Starting with the red (100) surface, we see that the reflectivity of the surfaces toward the (100) orientation decreases around 0.25% at the catalytic ignition as shown in panel a. This is attributed to the formation of the well-known 2–3 Å thick (*√*5× *√*5)*R*27° surface oxide which has been extensively described in the literature and has been found to form during both simple oxidation studies and during CO oxidation.^41−46^ The decrease in the reflectivity matches the expected oxide thickness.

Moving on to the blue (111) surface, which has been studied during CO oxidation at near-ambient pressures by Toyoshima et al.^47^ and in UHV by Zhang et al.^48^ In both studies, the surface is shown to form a Pd_5_O_4_ surface oxide.^49^ In our measurement, we see that the surfaces very close to the (111) surface lose around 0.3% reflectivity at the ignition which we attribute to the formation of this surface oxide.

In contrast, the green Pd(110) surface, which has been less studied, remains the brightest surface throughout the ignition. This suggests that no surface oxide is formed and that the reaction proceeds via the Langmuir–Hinshelwood mechanism. This lack of a surface oxide is consistent with the results of Westerström et al.^50^

The discussion of the surface oxide formation on the high-index Pd surfaces is more complex. We begin by looking at the (553) and (522) surfaces. At the ignition, the cyan (553) surface loses 0.25% reflectivity while the purple (522) loses around 0.1%, suggesting differences in surface oxide formation. The (553) surface is known to exhibit a complex behavior where the surface facets exhibit various length (111) terraces connected by (110) steps, resulting in (111) and (332) surface orientations. This is further complicated by the matter that the faceting seems to be different in pure oxidation and in CO oxidation.^51,52^ Looking at Figure 6c, we also see that the surfaces along the edge between the (111) and (110) directions vary in reflectivity in a rather sporadic fashion, further confirming the complexity of the oxygen-induced faceting of vicinal Pd surfaces. Some of the orientations exhibited by the refaceting may cause surface oxide formation, while others do not. The (112) surface, which is close to the (522) orientation, has also been shown to exhibit faceting.^53^ For the range of surfaces between the (100) and (110) surface orientations with B-type steps, we observe a gradually decreasing amount of surface oxides that coincides with the inability of the (110) surface to form a surface oxide.^50^

#### 3.2.2 Bulk Oxide Formation

As the sample is heated further, some grains exhibit a significant reduction in reflectivity, which we attribute to the formation of a thicker bulk oxide, as shown in Figure 7. We can again compare the results of this measurement with the literature, starting with low-index surfaces.

As the sample temperature is increased further, the reflectivity of the surfaces close to (100) begins to reduce significantly, which is attributed to the formation of a thicker bulk oxide. This has also been described in the literature.^41,42,45^ In a study by Goodwin et al. performed at similar gas conditions, this oxide was found to be around 70 Å thick,^54^ which is within the same order of magnitude as suggested by our measurement.

Moving on, we see that the grains close to the (110) orientation remain bright, suggesting that no bulk oxide is formed. This is consistent with the findings of Toyoshima et al., who studied Pd(110) during CO oxidation^55^ at 1 mbar. They found that the surface primarily remains covered in chemisorbed oxygen during the reaction with only small amounts of bulk oxide being formed even at high O_2_:CO ratios.

The surfaces close to (111) behave similarly, losing very little reflectance. This indicates that very little bulk oxide is formed after surface oxide formation, which is consistent with the results by Toyoshima et al. where only very small amounts of bulk oxide were observed.^56^

Moving on to bulk oxide formation on the high-index surfaces, we see that this is more consistent than surface oxide formation, with the entire B-edge between the (111) and (110) orientations remaining brighter, suggesting that it oxidizes considerably less than the rest of the sample surface.

Furthermore, it is apparent that there are different regions in the IPF that seem to behave similarly. For example, there is a very abrupt jump in reflectance between the (100) and (110) orientations. This could be attributed to a similar refaceting process occurring throughout each region, where longer terraces are connected by larger steps.

It is noteworthy that the darkest area is around the (210) orientation, where either significant refaceting occurs or significant amounts of bulk is formed. To our knowledge, no surfaces close to this orientation have been previously studied, making it difficult to attribute this effect to a particular surface behavior.

### 3.3 Step Density Parameter and Step Edge Parameter

We have also plotted the change in reflectivity of the grains in region a as a function of the SDP and SEP as introduced by Winkler et al.,^13^ as shown in Figure 8. Panel a shows the reflectivity of the grains as a function of the SDP, whereas panel b shows the reflectivity as a function of the SEP. We conclude that there is a linear correlation between the SDP and the sample reflectivity. The outliers are the grains close to the (110) orientation colored green. Concerning the SEP, we see no clear correlation.

**Figure 9:**
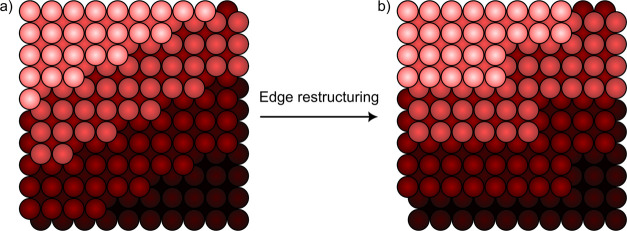
Edges in (a) can reconstruct into longer series of straight edges as shown in (b), which results in a similar step density, but a very different step edge structure. Thus, the SEP is more sensitive to reconstructions than the SDP.

In an attempt to explain this, we remind ourselves that the EBSD technique can determine only the bulk orientation of the crystal; the surface orientation indicated by EBSD then assumes a perfect cut of the bulk with no restructuring or faceting. This perfectly cut surface is then used to calculate the SEP and SDP. We speculate that difference in correlation between the SDP and SEP is because the SEP is more sensitive to surface restructuring as more complex edge structures are known to reconstruct into series of straight edges.^50,57^ This is illustrated in Figure 9. Thus, the actual SEP of the surface differs more to that indicated by EBSD than the actual SDP. Nonetheless, this data suggests that surfaces with higher step density oxidize more, even with a large sample size of probed surfaces.

#### 3.3.1 Catalytic Activity

A property of a catalyst that is perhaps more interesting than oxide formation is the catalytic activity itself, which we were unable to measure in this experiment. The question then is, does a correlation between oxide formation at the ignition and catalytic activity exist? Note that we are dealing with two separate phenomena here—the desorption of CO which prevents the catalytic reaction from occurring and the subsequent oxidation of the surface. In this work, we measure only the latter. This means that we do not necessarily expect a correlation between oxidation and activity. However, we could make the hypothesis that increased activity should result in more oxidizing CO-depleted gas conditions, which in turn results in increased oxide formation.

The activity of curved Pd crystals, which cover the region from the (553) orientation via (111) to the (322) orientation, has been investigated previously.^7,8,58^ Blomberg et al. show that the side with B-type steps becomes active before that with the A-type steps, which ignites before the (111) surface with no steps. This suggests that the (111) surface, which forms the thickest oxide layer (Figure 6c), is the least active. In the study by Vogel et al. where the low index surfaces were compared in their activity by using PEEM on a polycrystalline sample, this is also the case.^59^ They find the ignition is in the order (110)–(100)–(111), which in our case has the order thinnest to thickest oxide (brightest to darkest). Thus, perhaps unsurprisingly, there is no clear correlation between activity and oxide formation at the ignition.

#### 3.3.2 Gas Conditions

Another advantage of using polycrystals to perform surface science experiments is that using samples with grains that are small compared to the gas diffusion speed is one of few ways to really ensure all grains are exposed to nearly identical gas conditions throughout the experiment. Although the gases fed into the reactor can be accurately controlled using MFCs even when using single crystals, it is nearly impossible to correct for the fact that the active catalyst changes the gas environment.

## 4 Conclusions

In this work, we have demonstrated that the simple 2D-SOR technique can be used together with EBSD to map the reflectivity change and thus the oxidation of Pd as a function of surface orientation. While the reflectance data give less insight than direct measurements of the surface composition as done by XPS or HESXRD, the method presented in this work enables us to quickly measure a very large number of surface orientations in a single experiment and at high pressures.

This helps us to bridge both the materials and pressure gaps in catalysis and allows us to identify new regions of the surface orientation space for further exploration. For example, the abrupt change in reflectivity when moving along the edge between the (100) and (110) orientations is notable, as is the fact that the little-studied (310) orientation is the darkest and thus most likely the most oxidized surface orientation.

The application of the presented technique is also not limited to Pd. Any polycrystalline metal with the right grain size can be used as a sample. This means that a wide variety of metals can be probed to identify new surface orientations to study in more detail for use as potential catalysts. We thus think that performing reflectivity studies on transition metal polycrystals can have a large impact in bridging the materials gap in catalysis.

## Supporting Information Available

Video showing the reflectance change of the Pd sample during the entire experiment (Figure 5) (MP4)
